# Prevalence of Anemia among Children and Adolescents of Bangladesh: A Systematic Review and Meta-Analysis

**DOI:** 10.3390/ijerph20031786

**Published:** 2023-01-18

**Authors:** Shoumik Kundu, Sayeda Sadia Alam, Md Al-Tareq Mia, Tareq Hossan, Phil Hider, Md. Ibrahim Khalil, Kamarul Imran Musa, Md Asiful Islam

**Affiliations:** 1 Department of Biochemistry and Molecular Biology, Faculty of Biological Sciences, Jahangirnagar University, Savar, Dhaka 1342, Bangladesh; 2 Department of Bone Marrow Transplant, Washington University School of Medicine in St. Louis, St. Louis, MO 63110, USA; 3 Department of Population Health, University of Otago, Christchurch 8140, New Zealand; 4 Department of Community Medicine, School of Medical Sciences, Universiti Sains Malaysia, Kubang Kerian 16150, Malaysia; 5 Department of Haematology, School of Medical Sciences, Universiti Sains Malaysia, Kota Bharu 16150, Malaysia; 6 Institute of Metabolism and Systems Research, University of Birmingham, Birmingham B15 2TT, UK

**Keywords:** anemia, prevalence, Bangladesh, children, adolescents

## Abstract

The prevalence of anemia is high among children and adolescents in low- and middle-income countries because of undernutrition resulting from their poor socioeconomic status and lack of knowledge on proper nutrition. We conducted a systematic review and meta-analysis to determine the prevalence of anemia among children and adolescents aged between 6 months and 19 years in Bangladesh. Databases such as PubMed, Scopus, and Google Scholar were searched to identify the studies that reported the prevalence of anemia among children and adolescents. A total of 24 studies, including the data of 14,062 cases, were included in the systematic review and meta-analysis of the time period between 1997 and 2019. The random-effects model was used to calculate the summary estimates. The protocol was registered with PROSPERO (CRD42021246960). The pooled prevalence of anemia, iron deficiency anemia (IDA), and non-severe and severe anemia were 46.8% [95% CI: 36.0–57.6], 13.6% [95% CI: 8.0–19.2], 56.4% [95% CI: 39.6–73.1] and 0.7% [95% CI: 0.1–1.4], respectively. Prevalence of anemia exhibited the highest among the children aged ≤2 years. Briefly, 91.67% of the studies were of high quality. No significant publication bias was found; however, two outlier studies were detected. The prevalence of anemia among children and adolescents was estimated as high in Bangladesh.

## 1. Introduction

Anemia is a condition in which either the red blood cell count or the hemoglobin in blood is low, resulting in a reduction in oxygen-carrying capacity [1]. Iron deficiency is one of the major causes of anemia, accounting for approximately half of the world’s anemic population; however, anemia can also result from a lack of other micronutrients, such as riboflavin, vitamins A and B12, and folate [2,3]. A number of chronic diseases, such as tuberculosis, cancer, acquired immunodeficiency disease, and malaria, and other inherited or acquired disorders, such as thalassemia, may also lead to anemia [4,5,6]. Importantly, chronic diseases and infections are the second leading cause of anemia after iron deficiency. Previous shreds of evidence suggest that most people affected by viral, bacterial, or parasitic infections, cancer, autoimmune diseases (rheumatoid arthritis, systemic lupus erythematosus), and gastrointestinal disturbances (Inflammatory bowel syndrome) become anemic due to an elevated production of proinflammatory cytokines and free radicals, which damages erythroid progenitor cells [7]. According to the World Health Organization (WHO), children are the most vulnerable group to become anemic and the global prevalence of anemia among children aged from 6 to 59 months was estimated in 2011 to be 42.6%, with South-East Asia having the second highest prevalence (42.0%) after Africa (60.2%) [4]. A recent study in 2018 among more than 163,000 children aged between 6 and 59 months located in low- and middle-income countries (LMICs) reported that the prevalence of anemia was higher (55.8%) compared with developed countries, and severe anemia was present in 2.8% of participants [8]. Low socioeconomic status, maternal anemia, and poor sanitation are the main factors contributing to the high prevalence of childhood anemia in LMICs [9,10]. Females are more likely to be anemic in their adolescent years due to heavy menstruation and pregnancy, while micronutrient-deficient food and irregular food intake are the additional contributing factors among adolescents [11,12]. In addition, besides children and adolescents, prevention of anemia should be emphasized among pregnant and breastfeeding women, elderly persons, and people with chronic diseases, such as cancer and kidney disease [13].

Iron deficiency anemia (IDA) in young adults is often due to an iron-poor diet, medication interfering with iron absorption, eating disorders, and blood loss from menstruation [11]. Maternal food habits of ingesting iron-poor and less animal-based food sources are significant factors for IDA persisting at a high prevalence in pregnant women. Anemia among adolescents also has a major impact on normal physical ability and cognition [14]. Estimates of the prevalence of IDA among children and adolescents vary between 13.9 and 31.0% according to the recent studies conducted in Iran, Nepal, and Indonesia [15,16,17].

After exclusion of underlying medical conditions, mild-to-moderate iron deficiency anemia can be treated with iron supplements and micronutrient-enriched food. Acute severe anemia increases the risk of tachycardia, fatigue, hypotension, confusion and, infrequently, heart failure, among children [5]. Chronic anemia can result in bone fragility, liver and spleen enlargement, growth impairment, and decreased attention and motor activity [5].

Several strategies have been implemented and found to be useful to reduce anemia and IDA among children and adolescents, including iron, iron + folic acid, and multiple micronutrient supplementation, micronutrient-fortified complementary foods, zinc + iron + ascorbic acid fortified water, and promoting a diversified diet [18,19]. Reducing infections and parasitic diseases is also pivotal in addition to nutritional improvements and interventions, as those are the most likely factors to stimulate anemia after nutritional status [14].

Anemia is one of the most common and important hematological disorders to address with high priority among children and the adolescent population in Bangladesh. Therefore, we conducted this systematic review and meta-analysis to broadly categorize anemia in Bangladeshi children and adolescents according to the known causes and disease severity.

## 2. Methods

We followed PRISMA guidelines to conduct this systematic review and meta-analysis. We registered the study protocol with PROSPERO (CRD42021246960).

### 2.1. Eligibility Criteria

We included studies that reported the prevalence of anemia among young people aged from 6 months to 19 years old in Bangladesh without any language, sex, and time restrictions. We did not consider studies that examined anemia in groups of patients with previously known exposure because those exposures could interfere with the levels of hemoglobin (e.g., anemia among confirmed leukemia or thalassemia cases). We considered cross-sectional and cohort studies. We did not consider studies that did not present original quantitative results, such as review articles, opinions, case reports, commentaries, meeting abstracts, editorials, and theses.

### 2.2. Search Strategy

We developed a search strategy with related keywords associated with anemia, children, adolescents, and different locations in Bangladesh. Based on the appropriate keywords, we conducted advanced and expert searches in PubMed, Scopus, and Google Scholar databases up to 27 July 2022 (Table S1). We also explored references of the eligible studies to identify other potentially suitable studies. We entered references into EndNote X8 software and carefully deleted duplicate studies.

### 2.3. Study Selection

Three authors (S.K., S.S.A., and M.A.-T.M.) independently reviewed the titles, abstracts, and full texts of papers for eligibility, and settled any disagreements about inclusion by consensus.

### 2.4. Definitions and Data Extraction

We included the following four age categories: ≤2 years: toddlers; ≤5 years: pre-school children; <10 years: children; and 10 to ≤19: adolescents. We considered an individual anemic based on a measured hemoglobin concentration. For individuals aged <10 years, we used a hemoglobin concentration of 110 g/L as the cut-off value, and for individuals aged between 10 and 19 years, the cut-off value was 120 g/L. W employed a serum ferritin level of <12µg/L as the additional criterion to define cases of IDA [20,21]. We classified cases into two severity categories: 1) severe anemia: where the cut-off value of hemoglobin concentration was ≤70 g/L; 2) non-severe anemia: where the cut-off value of hemoglobin concentration was 71–109 g/L (for individuals aged <10 years) and 71–119 g/l (for individuals aged 10 to 19 years).

Three authors (S.K., S.S.A., and M.A.-T.M.) independently extracted data from each of the eligible studies, and another author (M.A.I.) reviewed any discrepancies and resolved by consensus. We extracted the following data from each eligible study into a Microsoft Excel spreadsheet: first author’s surname, year of publication, study design, study location, patient enrollment duration, number of patients, age of the participants, hemoglobin level measurement method, mean hemoglobin concentration, cut-off value of hemoglobin concentration, prevalence of anemia, prevalence of iron deficiency anemia, and prevalence of severe and non-severe anemia among different age groups.

### 2.5. Quality Assessment

Three authors (S.K., S.S.A., and M.A.I.) used the Joanna Briggs Institute (JBI) critical appraisal tools for cross-sectional and cohort studies to independently evaluate the quality of the included studies. We classified overall quality scores of ≤49, 50–70, and >70% as low-quality (high risk of bias), moderate-quality (moderate risk of bias), and high-quality (low risk of bias), respectively [22,23].

### 2.6. Data Analyses

We used a random-effects model to calculate the pooled prevalence with 95% confidence intervals (CIs). I^2^ statistics presented heterogeneity among the included studies (I^2^ > 75% indicating substantial heterogeneity); furthermore, we used Cochran’s Q test to determine the significance of the heterogeneity (we considered *p* < 0.05 statistically significant). We constructed funnel plots to detect publication bias; then, we performed Egger’s test. Next, we generated a Galbraith plot to identify any studies that were statistical outliers. We generated all analyses and plots using metaprop codes in meta (version 4.11–0) and metafor (version 2.4–0) packages of R (version 3.6.3) in RStudio (version 1.2.5033) software (RStudio, Inc., Boston, MA, USA) [24].

### 2.7. Subgroup and Sensitivity Analyses

We calculated the prevalence of both anemia and IDA across various age groups in subgroup analyses. In addition, we also determined the prevalence of severe and non-severe anemia in different age groups. For the sensitivity analyses, we excluded outlier, small (*n* < 200), and low- and moderate-quality studies.

## 3. Results

### 3.1. Study Selection

**Figure 1:**
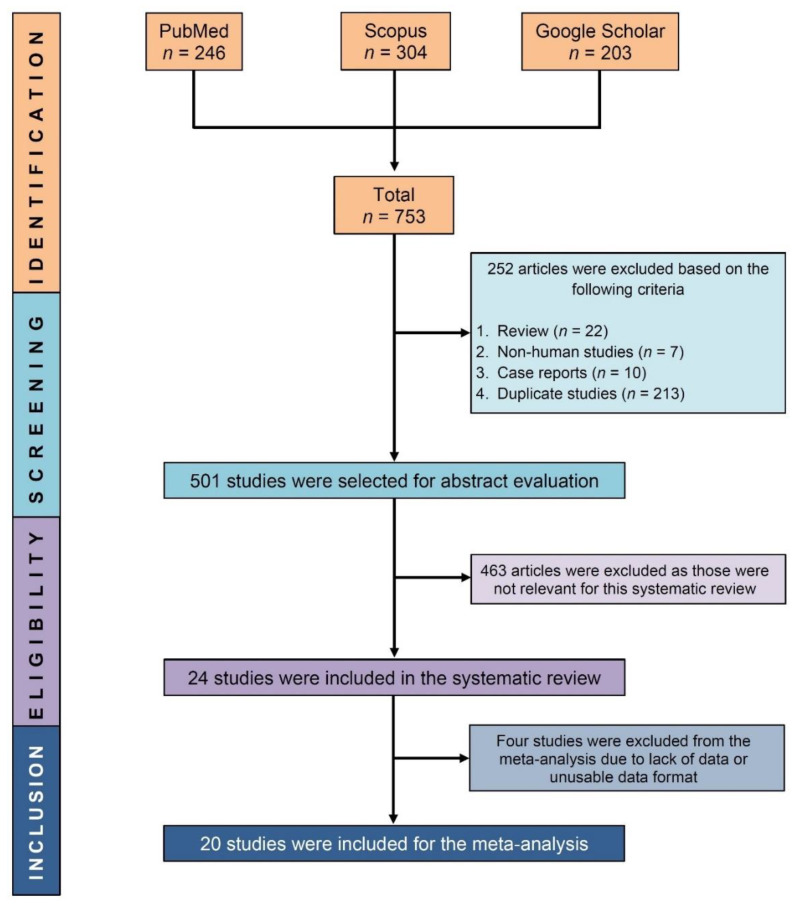
PRISMA flow diagram showing process of selecting eligible studies.

A total of 753 studies were identified using the search strategies. The abstracts and full texts of 501 studies were evaluated for eligibility after 252 papers were removed (213 duplicate studies, 22 review articles, 10 case reports, and 7 non-human studies). A total of 24 studies were found to be eligible for systematic review, and 20 of them were included in the meta-analysis (Figure 1).

### 3.2. Study Characteristics

A total of 14,062 cases were included in the systematic review, and the meta-analysis was conducted with 12,288 children and adolescents. The included studies were published from 1997 to 2019, covering 22 years. Details of the study characteristics are presented in Table 1.

### 3.3. Quality Assessment

Most (91.67%) studies were of high quality and exhibited a low risk of bias. A total of 8.33% of the studies were of moderate quality, presenting a moderate risk of bias. No study was found to be of low-quality and exhibiting a high risk of bias. (Tables S2 and S3).

### 3.4. Outcomes

**Figure 2:**
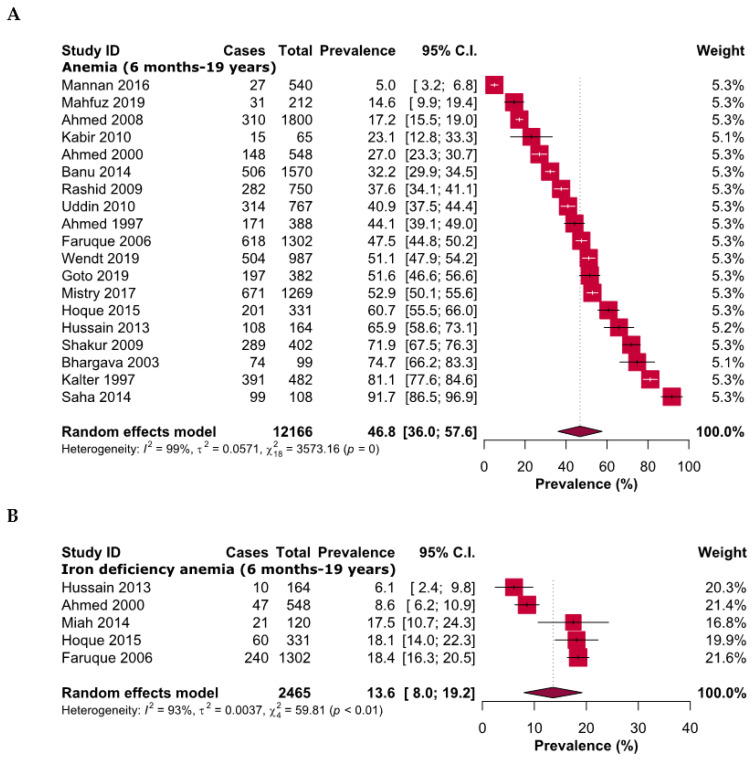
Prevalence of (**A**) anemia and (**B**) iron deficiency anemia in children and adolescents of Bangladesh.

**Figure 3:**
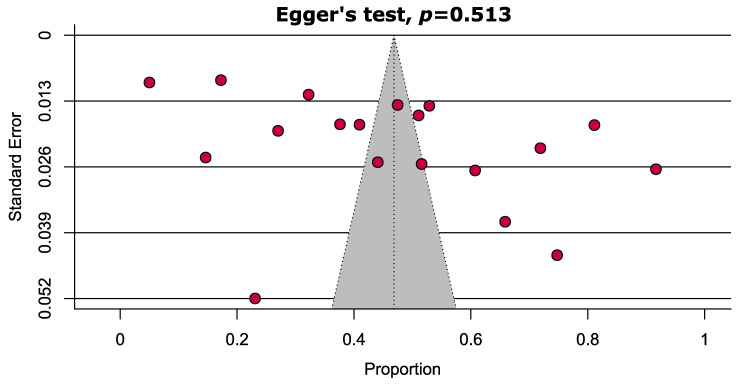
Funnel plot showing no evidence of publication bias estimating the prevalence of anemia in children and adolescents of Bangladesh.

**Figure 4:**
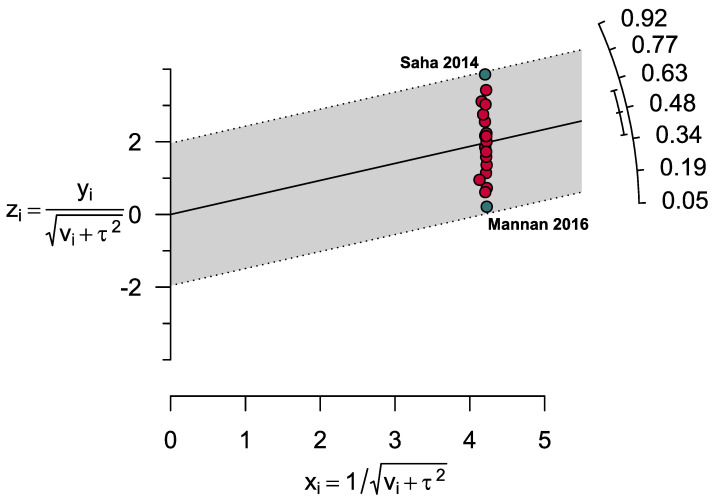
Galbraith plot representing two outlier studies estimating the prevalence of anemia in children and adolescents of Bangladesh.

Overall, the pooled prevalence of anemia and IDA was 46.8% [95% CI: 36.0–57.6] and 13.6% [95% CI: 8.0–19.2], respectively, among participants aged between 6 months and 19 years (Figure 2). From the subgroup analysis based on different age groups (≤2, ≤5, <10, and 10 to ≤19 years), the prevalence of anemia decreased with increasing age, except the children group (<10 years). The highest prevalence of anemia was reported among children aged ≤2 years (61.0%, [95% CI: 47.9–74.2]) and was the lowest amongst adolescents (42.1%, [95% CI: 28.5–55.7]) (Table 2 and Figure S1). The prevalence of anemia was quite similar among females and males aged from 6 months to 10 years, at 48.2% [95% CI: 35.9–50.5] and 50.4% [42.9–57.9], respectively; however, the prevalence was relatively lower for adolescent females (42.3%) [95% CI: 27.7–57.0]. The prevalence of IDA was the highest (18.1%, [95% CI: 14.0–22.3]) among preschool children ≤5 years and was the lowest amongst adolescents (12.5%, [95% CI: 5.9–19.1]); however, no study was available within the toddler age range. The prevalence of severe (1.0%, [95% CI: 0.0–2.0]) anemia was also consistently the highest among toddlers. (Table 2 and Figure S1). In another subgroup analysis based on the severity of anemia, the pooled prevalence of severe and non-severe anemia was 0.7% [95% CI: 0.1–1.4] and 56.4% [95% CI: 39.6–73.1], respectively (Table 2 and Figure S1). From the sensitivity analyses, it appears that the results are reliable; however, excluding studies with small sample sizes (n < 200) reduced the pooled prevalence by about 4.5%. (Table 3 and Figure S2). No obvious publication bias was evident in the funnel plot results (Figure 3), and two outlier studies were depicted from the Galbraith plot (Figure 4).

## 4. Discussion

Based on our findings in this meta-analysis, anemia is highly prevalent (46.8%) among Bangladeshi children and adolescents aged between 6 months and 19 years old. Maternal anemia, wealth, education status, and body mass index (BMI) were found to be the key factors contributing to anemia in children (<5 years) according to the Bangladesh Demographic Health Survey (BDHS) in 2011. In addition, children aged 6–12 months and 13–24 months showed a high prevalence (73.0%, and 70.0%, respectively) compared with children aged 49–59 months (38.0%) [47,48], which is consistent with our study, as toddlers depicted a 14.2% higher prevalence compared with the pooled prevalence between 6 months and 19 years. Lower maternal nutrition status, including anemia, makes children more vulnerable to stunting, wasting, and low birth weight. A global systematic analysis exhibited an overall mean rise of 6.0 g/l hemoglobin from 1995 to 2011 among children in South Asia aged 6–59 months; however, no changes were observed among pregnant women, and a 2.0 g/l increase was observed in non-pregnant women in the same region. Therefore, meeting the Global Nutrition Targets 2025 set by the WHO does not look realistic because, without improvements in the mother’s anemia status, managing childhood anemia is difficult [49,50]. A national survey conducted among 112714 children aged <5 years in India [51] showed a prevalence of 59.9%, which is much higher than in Bangladesh (47.3%), and identified vitamin A and iron deficiency as the main nutritional determinants. In Bangladesh, along with malnutrition, drinking-water sources are also a contributing factor to anemia, as childhood anemia was less prevalent in houses with a registered water supply, public standpipes, or a protected dug well [52]. A previous meta-analysis conducted in Ethiopia [53] among children and adolescents aged <14 years depicted a pooled prevalence of 34.4%, markedly lower than the prevalence in Bangladesh. Another study including toddlers aged from 6 to 23 months in Iran [54] demonstrated a higher prevalence (66.6%) of anemia compared to the prevalence of anemic toddlers in Bangladesh (61.0%).

Although two Ethiopian meta-analyses [53,55] among pre-school (<5 years) and school-children (5–17 years) represented an opposite correlation with increased age and anemia and showed about an 11.4% higher prevalence of anemia among preschool children compared with the school-going child, our study showed a slightly increased (3.5%) prevalence of anemia when the age limit increased from <5 years to <10 years. Intestinal parasitic infection was regarded as a significant determinant of anemia among school-aged children in Ethiopia [56]; however, the association between parasitic infection and risk of anemia among school-aged children was borderline and controversial, respectively, in studies conducted in Vietnam [57] and Bangladesh. Furthermore, Banu et al. [29] found a positive correlation between parasitic infection and anemia among Bangladeshi adolescents; however, Rahman et al. [58] found no significant correlation among school-aged children. Among school-aged children aged between 5 and 15 years in Bangalore, an intake of a vitamin A supplement and albendazole (an antiparasitic drug) as an intervention substantially prevented anemia [59].

In developed countries, IDA is an easily manageable condition with micronutrient supplements; however, a higher prevalence of chronic diseases, lead poisoning, and hemoglobinopathies make IDA more problematic for developing countries [60]. Iron-fortified foods globally serve as an important intervention to improve the status of IDA; however, iron supplementation exhibited mixed outcomes in improving the cognitive abilities of children. Micronutrient powders with substantial amounts of iron were also highly effective in reducing the prevalence of anemia in LMICs; however, the risk of intestinal inflammation should also be taken into consideration [59,60,61,62,63]. Obesity prevention among children and adolescents can be crucial to mitigating the chances of IDA, as obese children and adolescents are more vulnerable to depleted iron storage, which can lead to IDA [64]. A previous meta-analysis in Iran [15] illustrated an almost similar pooled prevalence (13.9%) of IDA among children and adolescents aged <18 years compared with our study (13.6%); however, another study in the same country [65] found a much-elevated proportion (27.7%) of IDA when the age limit was <6 years compared with the prevalence of IDA-affected children aged between 6 months and 5 years in Bangladesh (18.1%). Diagnostically, low serum iron levels and low transferrin saturation was found to be positively correlated with low Hb among IDA-affected adolescents in Bangladesh [20,21]. Besides Hb level and serum ferritin, some additional characteristics are also considered for defining IDA, including serum iron (<30 μg/dl), transferrin saturation (<10%), and mean corpuscular volume (70–75 fL). Moreover, low mean corpuscular volume (MCV) can result in microcytic anemia, where blood cells are smaller than the normal size, and microcytic anemia is also influenced by iron deficiency [66,67]. IDA-affected adolescents (>13 years) exhibited a significant risk of developing a plethora of psychiatric disorders, including bipolar and unipolar disorders, anxiety, tic disorder, developmental delay, and autistic spectrum and attention-deficit hyperactivity disorders, and the latter three were also common in IDA-affected children aged <13 years [68]. Therefore, adequate iron-rich food consumption to fulfill the demand for iron has no alternative in preventing persistent and irreversible neurological, psychological, and developmental impairments among both children and adolescents.

Among children, severe anemia often occurs due to an underlying medical condition, including malaria [69] and HIV exposure or HIV-positive status [70]. Girls with IDA who experience heavy menstrual bleeding during their adolescence are more vulnerable to severe anemia [71]. Two recent cross-sectional studies in Tamil Nadu, India [72], and Azad Jammu and Kashmir, Pakistan [73] found that about 10.48% and 5.7% of adolescents were severely anemic, respectively. These statistics are predominantly higher than the mean prevalence of severe anemia found through our meta-analysis, as well as each cross-sectional study that was subjected to the analysis. The highest prevalence of severe anemia was 2.4% in Bangladesh, where most of the severely anemic cases were affected by intestinal parasites [29].

A recent global estimation of anemia by Gardner et al. [74] presented that the prevalence of anemia among children <5 years is 7.1% higher than the global mean prevalence (39.7%); however, the prevalence of severe anemia was markedly lower in Bangladesh among the same subjects (0.7%) compared with 3.4% across the globe. Another meta-analysis study conducted in 2022 in Ethiopia [75] recorded a prevalence of 23.02% among adolescent girls aged 15–19 years and, compared with that, the prevalence of adolescent anemia among adolescents aged 10–19 years was remarkably high in Bangladesh (42.3%), representing an alarming scenario.

From the overall circumstances stated above, it can be understood that the prevalence of anemia among children and adolescents is noticeably higher compared with many regions in the world, and the following measures might help to improve the anemia situation in Bangladesh: (i) Improvement of maternal nutrition, education and wealth; (ii) accessibility to safe drinking water sources; (iii) providing iron supplements or fortified foods, micronutrient-rich foods, and supplements to both children and adolescents; (iv) timely prevention and treatment of parasitic and infectious diseases; and (v) preventing childhood and adolescent obesity.

Our study has several strengths. To the best of our knowledge, this is the first systematic review and meta-analysis based on the Bangladeshi child and adolescent population to estimate the prevalence of anemia and IDA among different age groups according to the degree of severity. This meta-analysis included an adequate number of studies to generate a robust outcome and represents a big portrait of the prevalence of anemia among both the child and adolescent population of Bangladesh.

However, the study has some limitations. Most of the previous studies were done in the Dhaka division, which covers almost 62.8% of the population in this study. If more studies took place on other divisions, we would be able to bring about a more comprehensive nationwide scenario. Some of our analyses also generated a substantial degree of heterogeneity. Furthermore, the prevalence of anemia among adolescents was only determined among females, and future studies should also be conducted on the male population. In addition, we had to consider only Hb and serum ferritin levels for defining IDA patients in meta-analysis; however, the estimation of serum iron, transferrin saturation, and MCV should be taken into diagnostic consideration.

## 5. Conclusions

Our study depicts a high prevalence of anemia and IDA among both children and the adolescent population in Bangladesh; however, the prevalence of severe anemia is low. The prevalence of anemia was high among children aged from 6 months to <10 years compared with adolescents aged from 10 to 19 years. Therefore, special emphasis should be given to children of early ages, providing them a balanced diet consisting of essential micronutrients, ensuring personal and familial hygiene, maternal education, and the safe reproductive health of adolescent girls to improve the overall status of anemia. Further studies should concentrate on determining the prevalence of IDA among Bangladeshi children because the number of existing studies is quite low. Again, most of the previous studies were done in the Dhaka division. Therefore, more studies should be conducted on other divisions. Moreover, nationwide studies can also be performed with a distinct sample size from each district to particularly focus on reducing the prevalence of anemia in highly affected areas in Bangladesh.

## Figures and Tables

**Table 1:** Major characteristics of included studies.

Study ID [References]	Study Design	Location	Patient Enrollment Time	Total Number of Hb Level Examined Patients (Girls)	Age (Range/Mean ± SD)	Hb Level Measurement Methods	Hb Concentration (Mean ± SD) (g/L)	Cut Off Value for Hb Concentration(g/L)
Adams 2017 [25]	Cohort	NR	September 2011 to November 2012	368 (171)	6 to 11 years	NR	124.4 ± 8.7	115
Ahmed 1997 [26]	Cross-sectional	Dhaka	December 1995 to January 1996	388 (388)	12 to 19 years	CMH method	119.7 ± 14.0	120
Ahmed 2000 [21]	Cross-sectional	Dhaka	September to November 1996	548 (548)	14.2 ± 1.3 years	CMH method	125.6 ± 11.6	120
Ahmed 2006 [27]	Cross-sectional	Dhaka	September to October 2000	381 (381)	11 to 16 years	CMH method	142.6 ± 13.2	11 years: 115 12–14 years: 120 15–16 years: 130
Ahmed 2008 [28]	Cross-sectional	Dhaka	March to May 2003	1800 (NR)	14 to 18 years	Using HA and CMH method	NR	120
Banu 2014 [29]	Cross-sectional	Dhaka	June 2006 to May 2009	1570 (1570)	10 to 19 years	Sahli’s method	NR	120
Bhargava 2003 [30]	Cross-sectional	Manikganj	June 1999 to May 2000	99 (60)	1 to 10 years	Using HA	115.3 ± 10.8	120
Faruque 2006 [31]	Cross-sectional	NR	October 1997 to December 1999	1302 (642)	24 to 72 months	CMH method	110.0 ±11.0	110
Goto 2019 [32]	Cohort	NR	2008 to 2016	382 (NR)	<5 years	Using HA	NR	110
Hoque 2015 [33]	Cross-sectional	Dhaka	June to August 2010	331 (126)	6 to 36 months	Using HA	NR	110
Hussain 2013 [20]	Cross-sectional	Dhaka	July 2011 to June 2012	164 (NR)	10 to 18 years	NR	108.0 ± 7.0	120
Kabir 2010 [34]	Cross-sectional	Dhaka	NR	65 (65)	15 to 19 years	Using commercial kits	128.0 ± 16.0	120
Kalter 1997 [35]	Cross-sectional	Dhaka	September 1994 to February 1995	482 (223)	2–60 months	Using HA	95.0 ± 19.0	110
Mahfuz 2019 [36]	Cohort	Dhaka	February 2010 to February 2017	212 (111)	5 years	Using HA	NR	110
Mannan 2016 [37]	Cohort	Dhaka	November 2001 to October 2003	540 (NR)	4.5 years	Using HA	128.6 ± 13.3	110
Miah 2014 [38]	Cross-sectional	Tangail	NR	120 (120)	12 to 17 years	Sahli’s method	NR	120
Mistry 2017 [39]	Cross-sectional	NR	October 2015 to January 2016	1269 (1269)	10 to 19 years	Using HA	118.2 ± 11.5	120
Persson 2000 [40]	Cross-sectional	Panchargarh and Thakurgaon	March to May 1998	164 (87)	6 to 12 years	Using HA	121.0 ± 10.0	115
Rashid 2009 [41]	Cross-sectional	Cumilla	September to December 2005	750 (553)	14.2 ± 3.2 years	Using HA	118.0 ± 15.9	Children: 110 Adolescent: 120
Saha 2014 [42]	Cross-sectional	Gazipur	March to June 2008	108 (108)	18 to 19 years	Hemoglobin color scale method	NR	120
Shahabuddin 2000 [43]	Cross-sectional	Narayanganj	1995	861 (456)	10 to 17 years	CMH method	NR	Male (10–15 years): 120 Male (15–19 years): 130 Female (10–15 years: 115 Female (15–19 years): 120
Shakur 2009 [44]	Cross-sectional	Gazipur	March 2005 to April 2007	402 (197)	6 months	Using HA	102.3 ± 12.4	110
Uddin 2010 [45]	Cross-sectional	Narayanganj	January to June 2010	767 (377)	6 to 59 months	Sahli’s method	109.0 ± 16.0	110
Wendt 2019 [46]	Cross-sectional	Sylhet	March to May 2015	987 (NR)	6 to 36 months	Using HA	108.0 ± 13.0	110

HA: Hemoglobin analyzer, CMH: Cyanmethemoglobin, Hb: Hemoglobin, SD: Standard deviation, NR: Not reported.

**Table 2:** Pooled prevalence of anemia in different subgroups of children and adolescents of Bangladesh.

Subgroups		Pooled Prevalence [95% CIs] (%)	Number of Studies Analyzed	Total Number of Patients	Heterogeneity	
					I^2^	*p*-Value
Anemia	6 months–2 years	61.0 [47.9–74.2]	3	1163	96%	<0.0001
	6 months–5 years	47.3 [27.0–67.7]	9	4498	100%	<0.0001
	6 months–10 years	50.8 [34.6–66.9]	12	6095	100%	<0.0001
	10–19 years	42.1 [28.5–55.7]	9	6267	99%	<0.0001
Iron deficiency anemia	6 months–5 years	18.1 [14.0–22.3]	1	331	NA	NA
	10–19 years	12.5 [5.9–19.1]	4	2134	95%	<0.0001
Severe anemia	6 months–19 years	0.7 [0.1–1.4]	7	4110	86%	<0.0001
	6 months–2 years	1.0 [0.0–2.0]	1	402	NA	NA
	6 months–5 years	0.7 [0.3–1.2]	3	1720	0%	0.56
	10–19 years	0.6 [0.0–1.8]	4	2390	92%	<0.0001
Non- severe anemia	6 months–19 years	56.4 [39.6–73.1]	7	4110	99%	<0.0001
	6 months–2 years	70.9 [66.5–75.3]	1	402	NA	NA
	6 months–5 years	60.2 [47.5–73.0]	3	1720	96%	<0.0001
	10–19 years	53.5 [26.2–80.8]	4	2390	99%	<0.0001
Anemia (Female)	6 months–10 years	48.2 [35.9–50.5]	3	1145	100%	<0.0001
	10–19 years	42.3 [27.7–57.0]	7	4303	99%	<0.0001
Anemia (Male)	6 months–10 years	50.4 [42.9–57.9]	3	1255	100%	<0.0001

CIs: Confidence intervals; NA: Not applicable.

**Table 3:** Sensitivity analyses.

Strategies of Sensitivity Analyses	Prevalence of Anemia (95% CIs) (%)	Difference of Pooled Prevalence Compared to the Main Result	Number of Studies Analyzed	Total Number Patients	Heterogeneity	
					I^2^	*p*-Value
Prevalence of anemia						
Excluding small studies (*n* < 200)	42.3 [31.0–53.7]	4.5% lower	15	11,730	100%	<0.0001
Excluding low- and moderate-quality studies	47.1 [34.9–59.2]	0.3% higher	16	10,635	100%	<0.0001
Excluding outlier studies	46.7 [37.3–56.1]	0.1% lower	17	11,518	99%	<0.0001
Prevalence of iron deficiency anemia						
Excluding small studies (*n* < 200)	15.0 [7.9–22.0]	1.4% higher	3	2181	95%	<0.0001
Excluding low- and moderate-quality studies	14.4 [7.1–21.7]	0.8% higher	3	999	89%	<0.0001
Excluding outlier studies	13.6 [8.0–19.2]	No changes	5	2465	93%	<0.0001

CIs: Confidence intervals.

## Data Availability

All data generated or analyzed during this study are included in this published article and supplementary materials.

## Supplementary Materials

The following supporting information can be downloaded at: https://www.mdpi.com/article/10.3390/ijerph20031786/s1, Figure S1: Subgroup analysis assessing the prevalence of anemia (**A**–**D**), iron deficiency anemia (**E**–**F**), severe anemia (**G**–**J**) and non-severe anemia (**K**–**N**) in different age groups of children and adolescents of Bangladesh; Figure S2: Sensitivity analysis assessing the prevalence of (**A**–**C**) anemia and (**D**–**F**) iron deficiency anemia in children and adolescents of Bangladesh; Table S1: Search strategies; Table S2: Quality assessment of the cross-sectional studies; Table S3: Quality assessment of the cohort studies.
