# Supplementary material for: Prevalence of Anemia among Children and Adolescents of Bangladesh: A Systematic Review and Meta-Analysis
Source: Int J Environ Res Public Health. 2023 Jan 18;20(3):1786. doi: 10.3390/ijerph20031786 (PMC9914578; doi:10.3390/ijerph20031786)
Supplement: Supplementary file 1 [file ijerph-20-01786-s001.zip › ijerph-1870331-supplementary.pdf]

A

Study ID	Cases	Total	Prevalence	95% C.I.	Weight
**Anemia (6 months-2 years)**					
Rashid 2009	194	395	49.1	[44.2; 54.0]	33.2%
Uddin 2010	227	366	62.0	[57.0; 67.0]	33.2%
Shakur 2009	289	402	71.9	[67.5; 76.3]	33.6%
**Random effects model**		**1163**	**61.0**	**[47.9; 74.2]**	**100.0%**

Heterogeneity: $I^2 = 96\%$, $\tau^2 = 0.0129$, $\chi^2_2 = 45.70$ ($p < 0.01$)

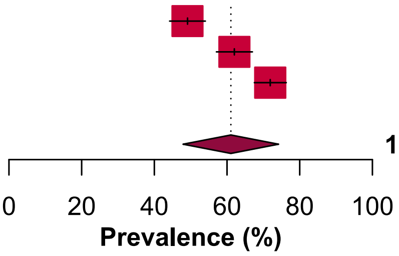

B

Study ID	Cases	Total	Prevalence	95% C.I.	Weight
**Anemia (6 months-5 years)**					
Mannan 2016	27	540	5.0	[3.2; 6.8]	11.2%
Mahfuz 2019	31	212	14.6	[9.9; 19.4]	11.1%
Uddin 2010	314	767	40.9	[37.5; 44.4]	11.1%
Rashid 2009	194	395	49.1	[44.2; 54.0]	11.1%
Wendt 2019	504	987	51.1	[47.9; 54.2]	11.1%
Goto 2019	197	382	51.6	[46.6; 56.6]	11.1%
Hoque 2015	201	331	60.7	[55.5; 66.0]	11.1%
Shakur 2009	289	402	71.9	[67.5; 76.3]	11.1%
Kalter 1997	391	482	81.1	[77.6; 84.6]	11.1%
**Random effects model**		**4498**	**47.3**	**[27.0; 67.7]**	**100.0%**

Heterogeneity: $I^2 = 100\%$, $\tau^2 = 0.0966$, $\chi^2_8 = 2312.03$ ($p = 0$)

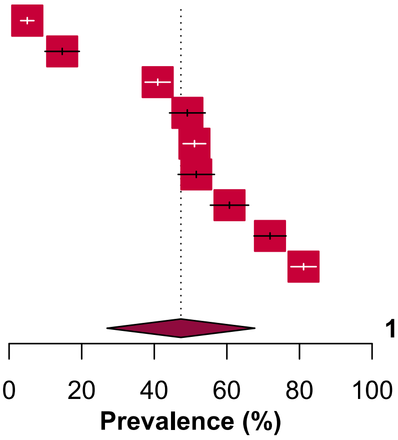

C

Study ID	Cases	Total	Prevalence	95% C.I.	Weight
**Anemia (6 months-less than 10 years)**					
Mannan 2016	27	540	5.0	[3.2; 6.8]	8.4%
Mahfuz 2019	31	212	14.6	[9.9; 19.4]	8.3%
Uddin 2010	314	767	40.9	[37.5; 44.4]	8.4%
Faruque 2006	618	1302	47.5	[44.8; 50.2]	8.4%
Rashid 2009	194	395	49.1	[44.2; 54.0]	8.3%
Wendt 2019	504	987	51.1	[47.9; 54.2]	8.4%
Goto 2019	197	382	51.6	[46.6; 56.6]	8.3%
Hoque 2015	201	331	60.7	[55.5; 66.0]	8.3%
Andersen 2016	121	196	61.7	[54.9; 68.5]	8.3%
Shakur 2009	289	402	71.9	[67.5; 76.3]	8.3%
Bhargava 2003	74	99	74.7	[66.2; 83.3]	8.2%
Kalter 1997	391	482	81.1	[77.6; 84.6]	8.4%
**Random effects model**		**6095**	**50.8**	**[34.6; 66.9]**	**100.0%**

Heterogeneity: $I^2 = 100\%$, $\tau^2 = 0.0810$, $\chi^2_{11} = 2498.19$ ($p = 0$)

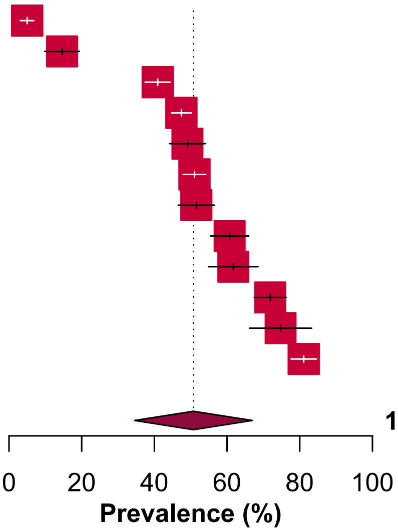

D

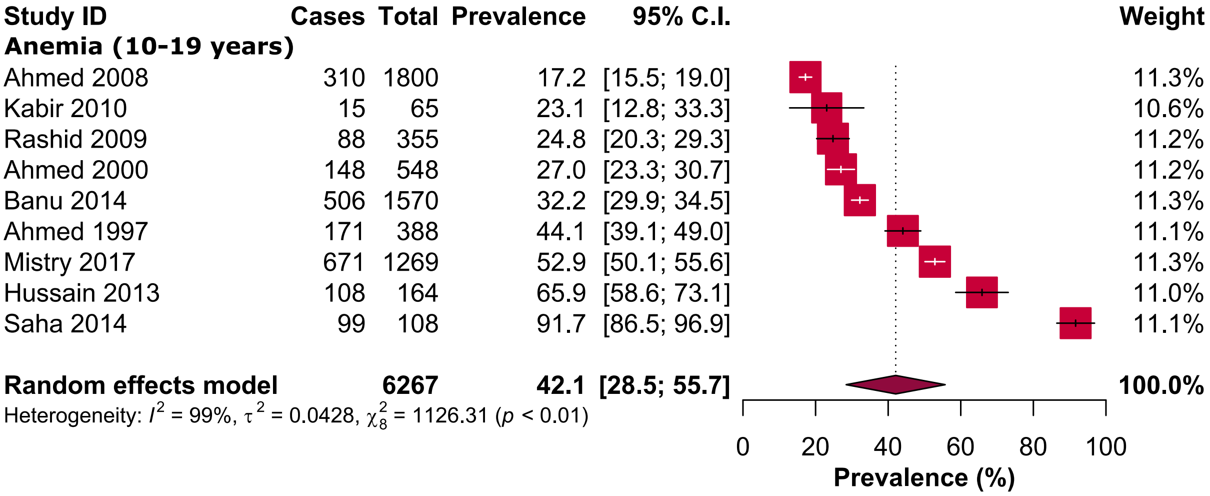

Study ID Cases Total Prevalence 95% C.I. Weight
Anemia (10-19 years)
Ahmed 2008 310 1800 17.2 [15.5; 19.0] 11.3%
Kabir 2010 15 65 23.1 [12.8; 33.3] 10.6%
Rashid 2009 88 355 24.8 [20.3; 29.3] 11.2%
Ahmed 2000 148 548 27.0 [23.3; 30.7] 11.2%
Banu 2014 506 1570 32.2 [29.9; 34.5] 11.3%
Ahmed 1997 171 388 44.1 [39.1; 49.0] 11.1%
Mistry 2017 671 1269 52.9 [50.1; 55.6] 11.3%
Hussain 2013 108 164 65.9 [58.6; 73.1] 11.0%
Saha 2014 99 108 91.7 [86.5; 96.9] 11.1%
Random effects model 6267 42.1 [28.5; 55.7] 100.0%
Heterogeneity: I^2 = 99%, τ^2 = 0.0428, χ^2_8 = 1126.31 (p < 0.01)
0 20 40 60 80 100
Prevalence (%)

E

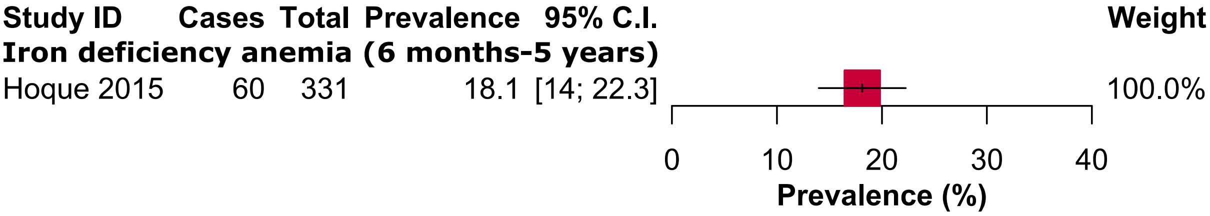

Study ID Cases Total Prevalence 95% C.I. Weight
Iron deficiency anemia (6 months-5 years)
Hoque 2015 60 331 18.1 [14; 22.3] 100.0%
0 10 20 30 40
Prevalence (%)

F

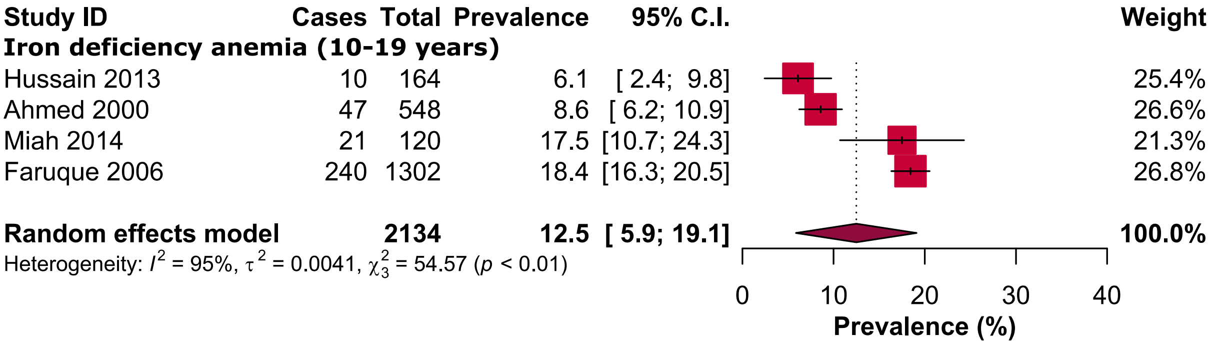

Study ID Cases Total Prevalence 95% C.I. Weight
Iron deficiency anemia (10-19 years)
Hussain 2013 10 164 6.1 [2.4; 9.8] 25.4%
Ahmed 2000 47 548 8.6 [6.2; 10.9] 26.6%
Miah 2014 21 120 17.5 [10.7; 24.3] 21.3%
Faruque 2006 240 1302 18.4 [16.3; 20.5] 26.8%
Random effects model 2134 12.5 [5.9; 19.1] 100.0%
Heterogeneity: I^2 = 95%, τ^2 = 0.0041, χ^2_3 = 54.57 (p < 0.01)
0 10 20 30 40
Prevalence (%)

G

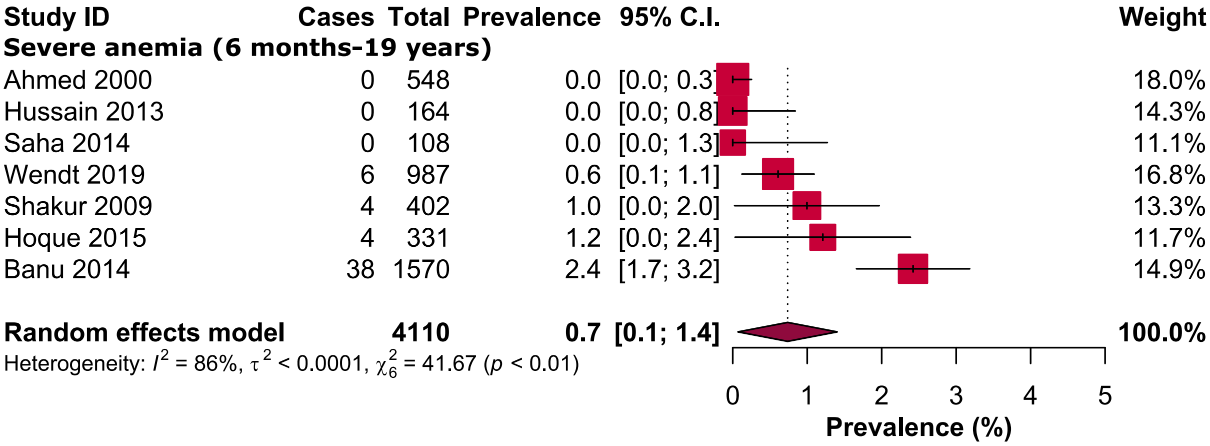

Study ID Cases Total Prevalence 95% C.I. Weight
Severe anemia (6 months-19 years)
Ahmed 2000 0 548 0.0 [0.0; 0.3] 18.0%
Hussain 2013 0 164 0.0 [0.0; 0.8] 14.3%
Saha 2014 0 108 0.0 [0.0; 1.3] 11.1%
Wendt 2019 6 987 0.6 [0.1; 1.1] 16.8%
Shakur 2009 4 402 1.0 [0.0; 2.0] 13.3%
Hoque 2015 4 331 1.2 [0.0; 2.4] 11.7%
Banu 2014 38 1570 2.4 [1.7; 3.2] 14.9%
Random effects model 4110 0.7 [0.1; 1.4] 100.0%
Heterogeneity: I^2 = 86%, τ^2 < 0.0001, χ^2_6 = 41.67 (p < 0.01)
0 1 2 3 4 5
Prevalence (%)

H

Study ID	Cases	Total	Prevalence	95% C.I.	Weight
**Severe anemia (6 months-2 years)**					
Shakur 2009	4	402	1.0	[0; 2]	100.0%

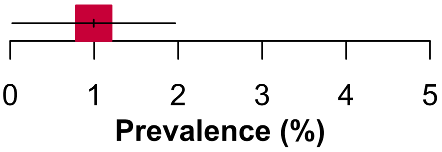

I

Study ID	Cases	Total	Prevalence	95% C.I.	Weight
**Severe anemia (6 months-5 years)**					
Wendt 2019	6	987	0.6	[0.1; 1.1]	70.4%
Shakur 2009	4	402	1.0	[0.0; 2.0]	17.6%
Hoque 2015	4	331	1.2	[0.0; 2.4]	12.0%
**Random effects model**		**1720**	**0.7**	**[0.3; 1.2]**	**100.0%**

Heterogeneity: $I^2 = 0\%$, $\tau^2 = 0$, $\chi^2_2 = 1.16$ ($p = 0.56$)

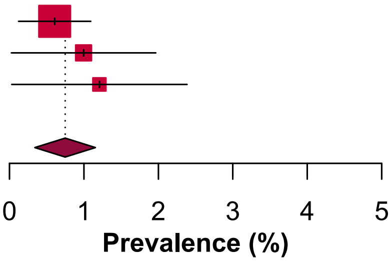

J

Study ID	Cases	Total	Prevalence	95% C.I.	Weight
**Severe anemia (10-19 years)**					
Ahmed 2000	0	548	0.0	[0.0; 0.3]	28.1%
Hussain 2013	0	164	0.0	[0.0; 0.8]	24.9%
Saha 2014	0	108	0.0	[0.0; 1.3]	21.5%
Banu 2014	38	1570	2.4	[1.7; 3.2]	25.5%
**Random effects model**		**2390**	**0.6**	**[0.0; 1.8]**	**100.0%**

Heterogeneity: $I^2 = 92\%$, $\tau^2 = 0.0001$, $\chi^2_3 = 35.48$ ($p < 0.01$)

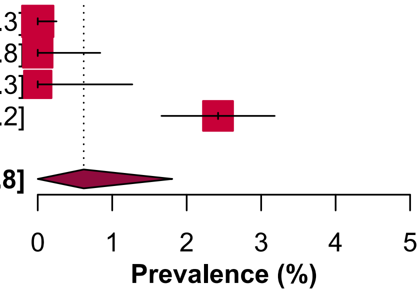

K

Study ID	Cases	Total	Prevalence	95% C.I.	Weight
**Non-severe anemia (6 months-19 years)**					
Ahmed 2000	148	548	27.0	[23.3; 30.7]	14.3%
Banu 2014	468	1570	29.8	[27.5; 32.1]	14.4%
Wendt 2019	498	987	50.5	[47.3; 53.6]	14.4%
Hoque 2015	197	331	59.5	[54.2; 64.8]	14.2%
Hussain 2013	108	164	65.9	[58.6; 73.1]	14.1%
Shakur 2009	285	402	70.9	[66.5; 75.3]	14.3%
Saha 2014	99	108	91.7	[86.5; 96.9]	14.2%
**Random effects model**		**4110**	**56.4**	**[39.6; 73.1]**	**100.0%**

Heterogeneity: $I^2 = 99\%$, $\tau^2 = 0.0504$, $\chi^2_6 = 773.70$ ($p < 0.01$)

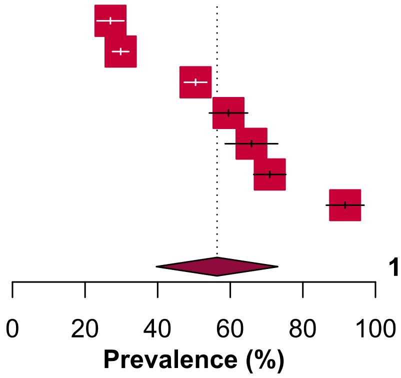

L

Study ID	Cases	Total	Prevalence	95% C.I.	Weight
**Non-severe anemia (6 months-2 years)**					
Shakur 2009	285	402	70.9	[66.5; 75.3]	100.0%

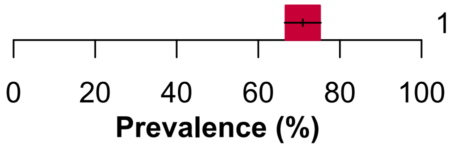

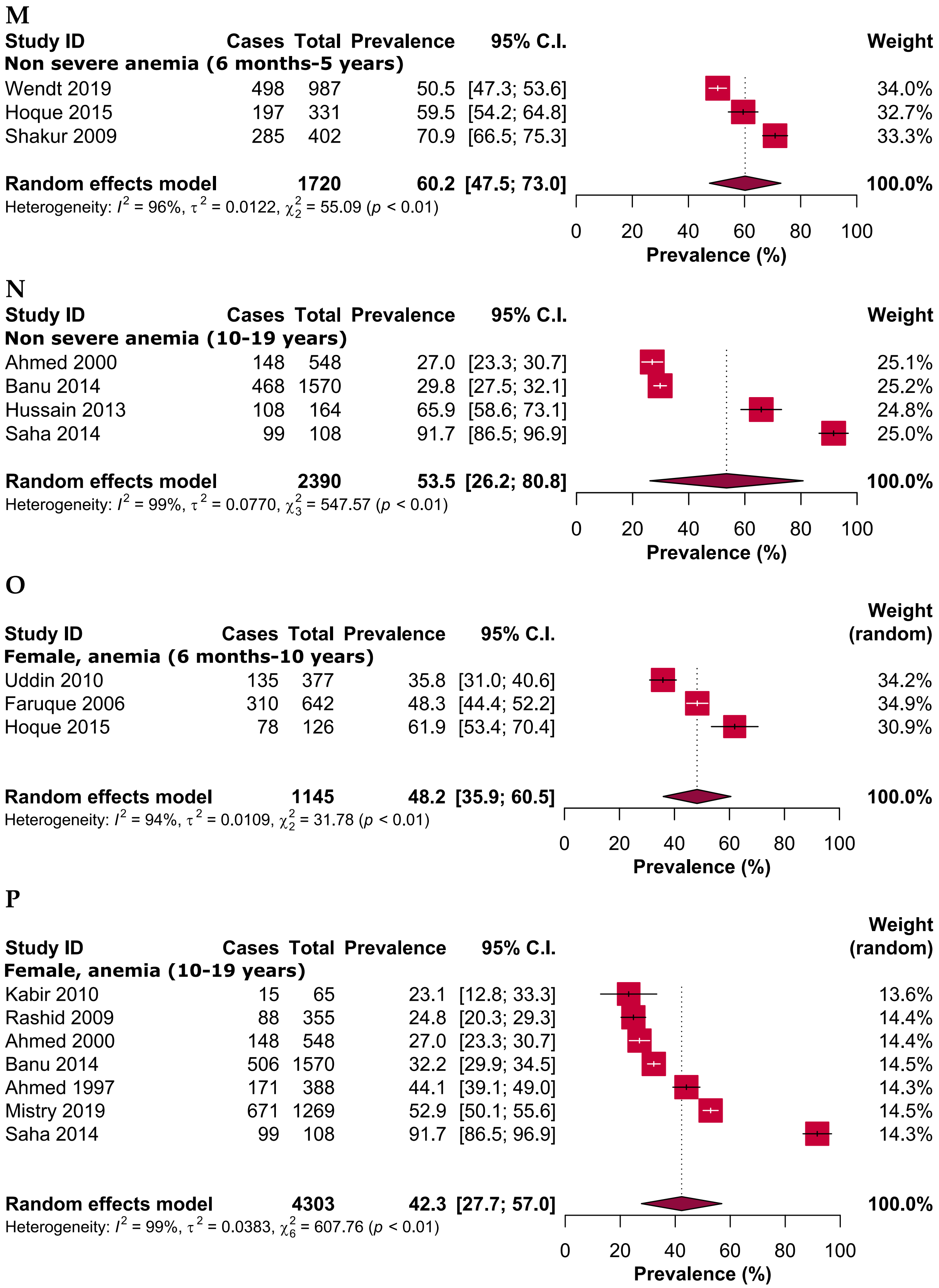
M
Study ID Cases Total Prevalence 95% C.I. Weight
Non severe anemia (6 months-5 years)
Wendt 2019 498 987 50.5 [47.3; 53.6] 34.0%
Hoque 2015 197 331 59.5 [54.2; 64.8] 32.7%
Shakur 2009 285 402 70.9 [66.5; 75.3] 33.3%
Random effects model 1720 60.2 [47.5; 73.0] 100.0%
Heterogeneity: I^2 = 96%, τ^2 = 0.0122, χ^2_2 = 55.09 (p < 0.01)
0 20 40 60 80 100
Prevalence (%)
N
Study ID Cases Total Prevalence 95% C.I. Weight
Non severe anemia (10-19 years)
Ahmed 2000 148 548 27.0 [23.3; 30.7] 25.1%
Banu 2014 468 1570 29.8 [27.5; 32.1] 25.2%
Hussain 2013 108 164 65.9 [58.6; 73.1] 24.8%
Saha 2014 99 108 91.7 [86.5; 96.9] 25.0%
Random effects model 2390 53.5 [26.2; 80.8] 100.0%
Heterogeneity: I^2 = 99%, τ^2 = 0.0770, χ^2_3 = 547.57 (p < 0.01)
0 20 40 60 80 100
Prevalence (%)
O
Study ID Cases Total Prevalence 95% C.I. Weight (random)
Female, anemia (6 months-10 years)
Uddin 2010 135 377 35.8 [31.0; 40.6] 34.2%
Faruque 2006 310 642 48.3 [44.4; 52.2] 34.9%
Hoque 2015 78 126 61.9 [53.4; 70.4] 30.9%
Random effects model 1145 48.2 [35.9; 60.5] 100.0%
Heterogeneity: I^2 = 94%, τ^2 = 0.0109, χ^2_2 = 31.78 (p < 0.01)
0 20 40 60 80 100
Prevalence (%)
P
Study ID Cases Total Prevalence 95% C.I. Weight (random)
Female, anemia (10-19 years)
Kabir 2010 15 65 23.1 [12.8; 33.3] 13.6%
Rashid 2009 88 355 24.8 [20.3; 29.3] 14.4%
Ahmed 2000 148 548 27.0 [23.3; 30.7] 14.4%
Banu 2014 506 1570 32.2 [29.9; 34.5] 14.5%
Ahmed 1997 171 388 44.1 [39.1; 49.0] 14.3%
Mistry 2019 671 1269 52.9 [50.1; 55.6] 14.5%
Saha 2014 99 108 91.7 [86.5; 96.9] 14.3%
Random effects model 4303 42.3 [27.7; 57.0] 100.0%
Heterogeneity: I^2 = 99%, τ^2 = 0.0383, χ^2_6 = 607.76 (p < 0.01)
0 20 40 60 80 100
Prevalence (%)

Q

Study ID	Cases	Total	Prevalence	95% C.I.	Weight (random)
**Male, anemia (6 months-10 years)**					
Uddin 2010	179	390	45.9	[41.0; 50.8]	33.9%
Faruque 2006	308	660	46.7	[42.9; 50.5]	36.0%
Hoque 2015	123	205	60.0	[53.3; 66.7]	30.1%
**Random effects model**		**1255**	**50.4**	**[42.9; 57.9]**	**100.0%**

Heterogeneity: $I^2 = 85\%$, $\tau^2 = 0.0037$, $\chi^2_2 = 13.24$ ($p < 0.01$)

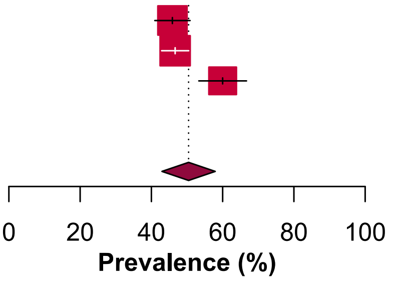

Figure S1. Subgroup analysis assessing the prevalence of anemia (A-D), iron deficiency anemia (E-F), severe anemia (G-J), non-severe anemia (K-N) in different age groups and gender (O-Q) of children and adolescents of Bangladesh.

A

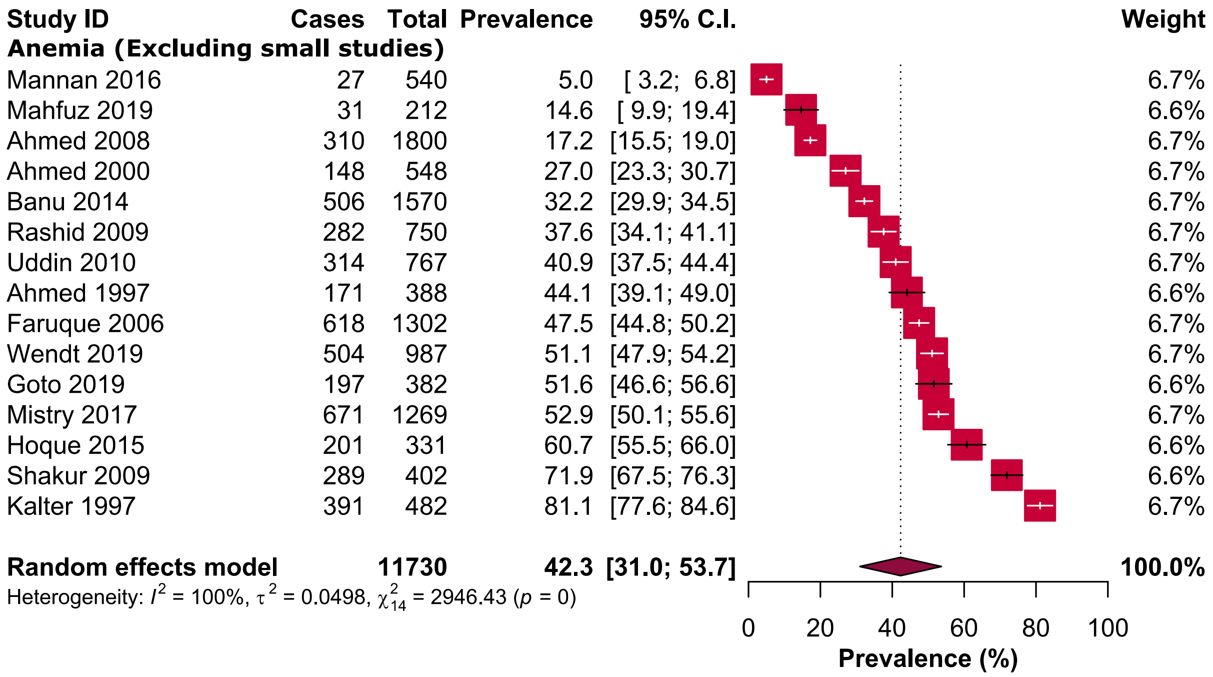
Study ID Cases Total Prevalence 95% C.I. Weight
Anemia (Excluding small studies)
Mannan 2016 27 540 5.0 [3.2; 6.8] 6.7%
Mahfuz 2019 31 212 14.6 [9.9; 19.4] 6.6%
Ahmed 2008 310 1800 17.2 [15.5; 19.0] 6.7%
Ahmed 2000 148 548 27.0 [23.3; 30.7] 6.7%
Banu 2014 506 1570 32.2 [29.9; 34.5] 6.7%
Rashid 2009 282 750 37.6 [34.1; 41.1] 6.7%
Uddin 2010 314 767 40.9 [37.5; 44.4] 6.7%
Ahmed 1997 171 388 44.1 [39.1; 49.0] 6.6%
Faruque 2006 618 1302 47.5 [44.8; 50.2] 6.7%
Wendt 2019 504 987 51.1 [47.9; 54.2] 6.7%
Goto 2019 197 382 51.6 [46.6; 56.6] 6.6%
Mistry 2017 671 1269 52.9 [50.1; 55.6] 6.7%
Hoque 2015 201 331 60.7 [55.5; 66.0] 6.6%
Shakur 2009 289 402 71.9 [67.5; 76.3] 6.6%
Kalter 1997 391 482 81.1 [77.6; 84.6] 6.7%
Random effects model 11730 42.3 [31.0; 53.7] 100.0%
Heterogeneity: I^2 = 100%, τ^2 = 0.0498, χ^2_{14} = 2946.43 (p = 0)
0 20 40 60 80 100
Prevalence (%)

B

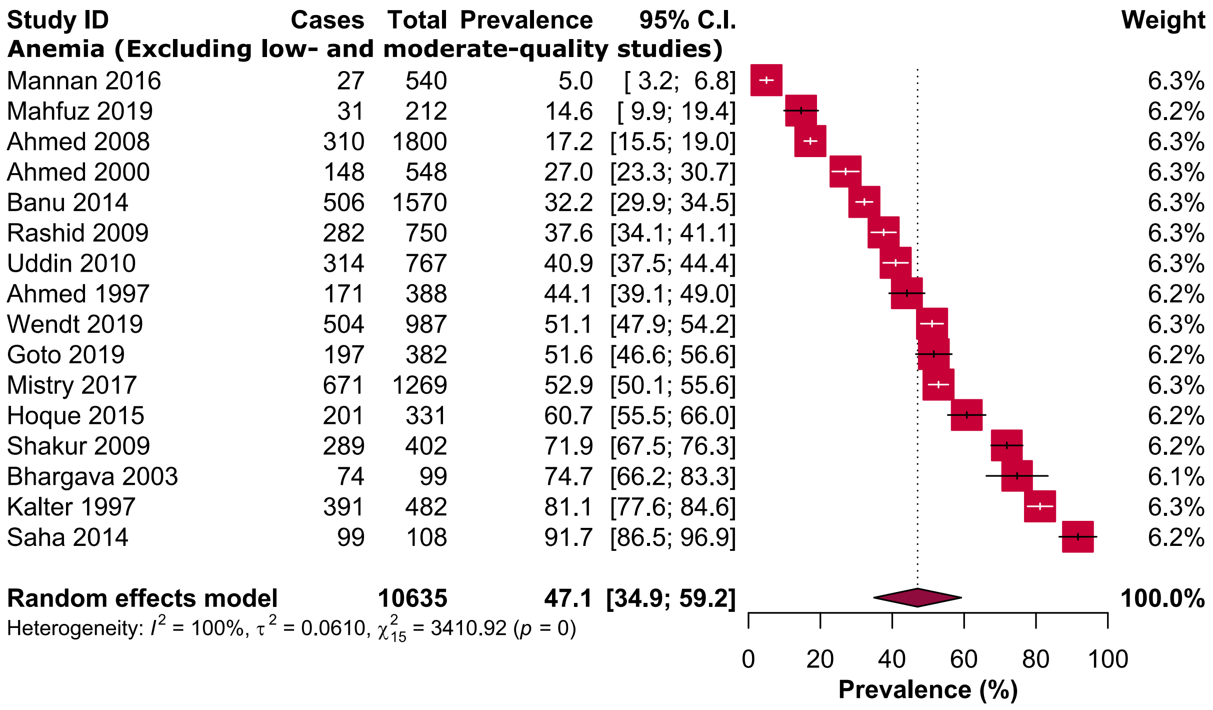
Study ID Cases Total Prevalence 95% C.I. Weight
Anemia (Excluding low- and moderate-quality studies)
Mannan 2016 27 540 5.0 [3.2; 6.8] 6.3%
Mahfuz 2019 31 212 14.6 [9.9; 19.4] 6.2%
Ahmed 2008 310 1800 17.2 [15.5; 19.0] 6.3%
Ahmed 2000 148 548 27.0 [23.3; 30.7] 6.3%
Banu 2014 506 1570 32.2 [29.9; 34.5] 6.3%
Rashid 2009 282 750 37.6 [34.1; 41.1] 6.3%
Uddin 2010 314 767 40.9 [37.5; 44.4] 6.3%
Ahmed 1997 171 388 44.1 [39.1; 49.0] 6.2%
Wendt 2019 504 987 51.1 [47.9; 54.2] 6.3%
Goto 2019 197 382 51.6 [46.6; 56.6] 6.2%
Mistry 2017 671 1269 52.9 [50.1; 55.6] 6.3%
Hoque 2015 201 331 60.7 [55.5; 66.0] 6.2%
Shakur 2009 289 402 71.9 [67.5; 76.3] 6.2%
Bhargava 2003 74 99 74.7 [66.2; 83.3] 6.1%
Kalter 1997 391 482 81.1 [77.6; 84.6] 6.3%
Saha 2014 99 108 91.7 [86.5; 96.9] 6.2%
Random effects model 10635 47.1 [34.9; 59.2] 100.0%
Heterogeneity: I^2 = 100%, τ^2 = 0.0610, χ^2_{15} = 3410.92 (p = 0)
0 20 40 60 80 100
Prevalence (%)

C

Study ID	Cases	Total	Prevalence	95% C.I.	Weight
**Anemia (Excluding outlier studies)**					
Mahfuz 2019	31	212	14.6	[9.9; 19.4]	5.9%
Ahmed 2008	310	1800	17.2	[15.5; 19.0]	6.0%
Kabir 2010	15	65	23.1	[12.8; 33.3]	5.6%
Ahmed 2000	148	548	27.0	[23.3; 30.7]	5.9%
Banu 2014	506	1570	32.2	[29.9; 34.5]	6.0%
Rashid 2009	282	750	37.6	[34.1; 41.1]	5.9%
Uddin 2010	314	767	40.9	[37.5; 44.4]	5.9%
Ahmed 1997	171	388	44.1	[39.1; 49.0]	5.9%
Faruque 2006	618	1302	47.5	[44.8; 50.2]	6.0%
Wendt 2019	504	987	51.1	[47.9; 54.2]	5.9%
Goto 2019	197	382	51.6	[46.6; 56.6]	5.9%
Mistry 2017	671	1269	52.9	[50.1; 55.6]	6.0%
Hoque 2015	201	331	60.7	[55.5; 66.0]	5.9%
Hussain 2013	108	164	65.9	[58.6; 73.1]	5.8%
Shakur 2009	289	402	71.9	[67.5; 76.3]	5.9%
Bhargava 2003	74	99	74.7	[66.2; 83.3]	5.7%
Kalter 1997	391	482	81.1	[77.6; 84.6]	5.9%
**Random effects model**		**11518**	**46.7**	**[37.3; 56.1]**	**100.0%**

Heterogeneity: $I^2 = 99\%$, $\tau^2 = 0.0387$, $\chi^2_{16} = 1958.49$ ($p = 0$)

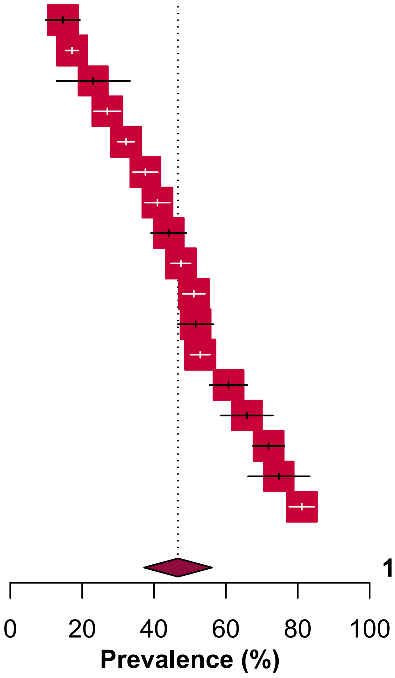

D

Study ID	Cases	Total	Prevalence	95% C.I.	Weight
**Iron deficiency anemia (Excluding small studies)**					
Ahmed 2000	47	548	8.6	[6.2; 10.9]	34.1%
Hoque 2015	60	331	18.1	[14.0; 22.3]	31.6%
Faruque 2006	240	1302	18.4	[16.3; 20.5]	34.3%
**Random effects model**		**2181**	**15.0**	**[7.9; 22.0]**	**100.0%**

Heterogeneity: $I^2 = 95\%$, $\tau^2 = 0.0036$, $\chi^2_{2} = 40.85$ ($p < 0.01$)

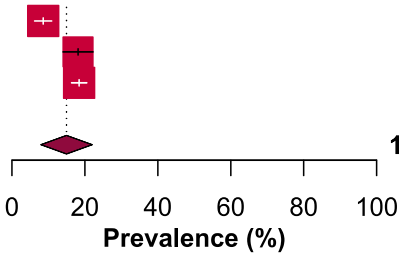

E

Study ID	Cases	Total	Prevalence	95% C.I.	Weight
**Iron deficiency anemia (Excluding low- and moderate-quality studies)**					
Ahmed 2000	47	548	8.6	[6.2; 10.9]	37.0%
Miah 2014	21	120	17.5	[10.7; 24.3]	28.8%
Hoque 2015	60	331	18.1	[14.0; 22.3]	34.2%
**Random effects model**		**999**	**14.4**	**[7.1; 21.7]**	**100.0%**

Heterogeneity: $I^2 = 89\%$, $\tau^2 = 0.0036$, $\chi^2_{2} = 18.76$ ($p < 0.01$)

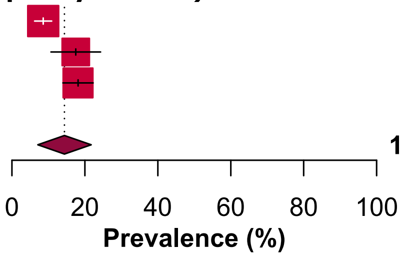

F

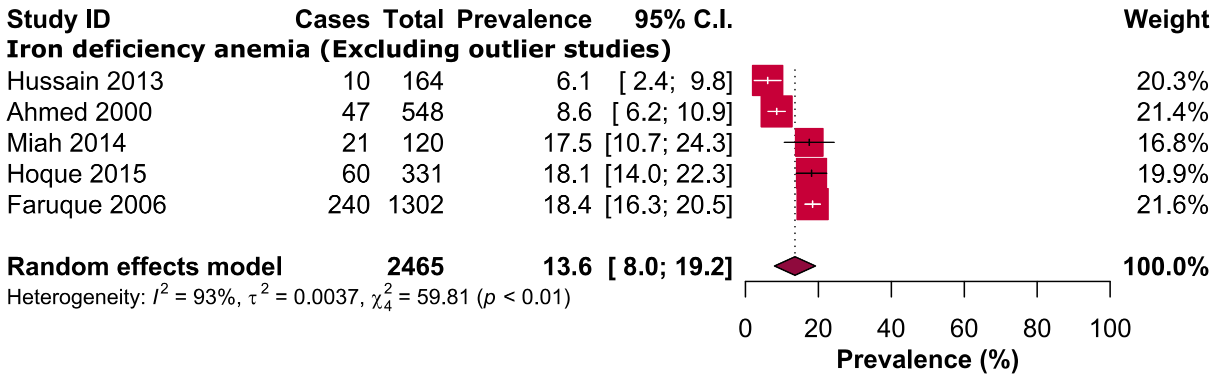

Figure S2. Sensitivity analysis assessing the prevalence of (A-C) anemia and (D-F) iron deficiency anemia in children and adolescents of Bangladesh.

Table S1. Search strategies

Databases	Search strategies
**PubMed**	((Anemia[Title/Abstract] OR Anaemia[Title/Abstract]) AND (Child* OR Adolescen* OR Pediatric* OR Paediatric*)) AND (Bangladesh OR Dhaka OR Chittagong OR Chattogram OR Rajshahi OR Rangpur OR Barisal OR Barishal OR Sylhet OR Khulna OR Mymensingh OR Dinajpur OR Bogra OR Bogura OR Comilla OR Cumilla OR Faridpur OR Pabna OR Noakhali OR "Cox's Bazar" OR Jessore OR Jashore OR Satkhira OR Gazipur OR Kushtia OR Sirajganj OR Gopalganj OR Jamalpur OR Gazipur OR Tangail OR Manikganj OR Patuakhali OR Rangamati OR Chandpur OR Netrakona OR Magura OR Naogaon OR Nilphamari)
**Scopus**	TITLE-ABS-KEY(Anemia OR Anaemia) AND TITLE-ABS-KEY(Child* OR Adolescen* OR Pediatric* OR Paediatric*) AND TITLE-ABS-KEY(Bangladesh OR Dhaka OR Chittagong OR Chattogram OR Rajshahi OR Rangpur OR Barisal OR Barishal OR Sylhet OR Khulna OR Mymensingh OR Dinajpur OR Bogra OR Bogura OR Comilla OR Cumilla OR Faridpur OR Pabna OR Noakhali OR "Cox's Bazar" OR Jessore OR Jashore OR Satkhira OR Gazipur OR Kushtia OR Sirajganj OR Gopalganj OR Jamalpur OR Gazipur OR Tangail OR Manikganj OR Patuakhali OR Rangamati OR Chandpur OR Netrakona OR Magura OR Naogaon OR Nilphamari)
**Google Scholar**	allintitle: (Anemia OR Anaemia) (Bangladesh OR Dhaka OR Chittagong OR Chattogram OR Rajshahi OR Rangpur OR Barisal OR Barishal OR Sylhet OR Khulna OR Mymensingh OR Dinajpur OR Bogra OR Bogura OR Comilla OR Cumilla OR Faridpur OR Pabna OR Noakhali OR "Cox's Bazar" OR Jessore OR Jashore OR Satkhira OR Gazipur OR Kushtia OR Sirajganj OR Gopalganj OR Jamalpur OR Gazipur OR Tangail OR Manikganj OR Patuakhali OR Rangamati OR Chandpur OR Netrakona OR Magura OR Naogaon OR Nilphamari)

Table S2. Quality assessment of the cross-sectional studies

No	Study ID	Critical appraisal checklist								Yes (%)
		1	2	3	4	5	6	7	8	
1	Ahmed 1997	Y	Y	N	Y	Y	Y	Y	Y	87.5
2	Ahmed 2000	Y	Y	Y	Y	Y	Y	Y	Y	100.0
3	Ahmed 2006	Y	Y	Y	Y	Y	Y	Y	Y	100.0
4	Ahmed 2008	Y	Y	N	Y	Y	Y	Y	Y	87.5
5	Banu 2014	Y	Y	Y	Y	N	N	Y	Y	75.0
6	Begum 2017	Y	Y	N	N	N	N	Y	U	37.5
7	Bhargava 2003	Y	Y	Y	Y	N	N	Y	Y	75.0
8	Faruque 2006	Y	Y	N	Y	N	N	Y	Y	62.5
9	Hoque 2015	Y	Y	Y	Y	Y	Y	Y	Y	100.0
10	Hussain 2013	Y	Y	N	N	N	N	Y	Y	50.0
11	Kabir 2010	Y	Y	N	Y	N	N	Y	Y	62.5
12	Kalter 1997	Y	Y	Y	Y	N	N	Y	Y	75.0
13	Miah 2014	Y	Y	Y	Y	N	N	Y	Y	75.0
14	Mistry 2017	Y	Y	Y	Y	Y	Y	Y	Y	100.0
15	Persson 2000	Y	Y	U	Y	Y	Y	Y	Y	87.5
16	Rashid 2009	Y	Y	Y	Y	Y	Y	Y	Y	100.0
17	Saha 2014	Y	Y	Y	Y	N	N	Y	Y	75.0
18	Shahabuddin 2000	Y	Y	Y	Y	U	N	Y	Y	75.0
19	Shakur 2009	Y	Y	Y	Y	Y	Y	Y	Y	100.0
20	Uddin 2010	Y	Y	Y	Y	N	N	Y	Y	75.0
21	Wendt 2019	Y	Y	Y	Y	Y	Y	Y	Y	100.0

1. Were the criteria for inclusion in the sample clearly defined? 2. Were the study subjects and the setting described in detail? 3. Was the exposure measured in a valid and reliable way? 4. Were objective, standard criteria used for measurement of the condition? 5. Were confounding factors identified? 6. Were strategies to deal with confounding factors stated? 7. Were the outcomes measured in a valid and reliable way? 8. Was appropriate statistical analysis used? Y: Yes, N: No, U: Unclear.

Table S3. Quality assessment of the cohort studies

No	Study ID	Critical appraisal checklist											Yes (%)
		**1**	**2**	**3**	**4**	**5**	**6**	**7**	**8**	**9**	**10**	**11**	
1	Adams 2017	Y	Y	Y	Y	Y	Y	Y	Y	N	N	Y	81.8
2	Goto 2019	Y	Y	Y	Y	Y	Y	Y	Y	Y	NA	Y	100.0
3	Mahfuz 2019	Y	Y	Y	Y	Y	Y	Y	Y	Y	NA	Y	100.0
4	Mannan 2016	Y	Y	Y	Y	Y	Y	Y	Y	Y	NA	Y	100.0

1. Were the two groups similar and recruited from the same population? 2. Were the exposures measured similarly to assign people 3. to both exposed and unexposed groups? 4. Was the exposure measured in a valid and reliable way? 5. Were confounding factors identified? 6. Were strategies to deal with confounding factors stated? 7. Were the groups/participants free of the outcome at the start of the study (or at the moment of exposure)? 8. Were the outcomes measured in a valid and reliable way? 9. Was the follow up time reported and sufficient to be long enough for outcomes to occur? 10. Was follow up complete, and if not, were the reasons to loss to follow up described and explored? 11. Were strategies to address incomplete follow up utilized? 12. Was appropriate statistical analysis used? Y: Yes, N: No, NA: Not applicable
